# ENLIGHTENme: Translating the Benefits of Self‐Administered Daytime Light Supplementation to Older Adults in the Real World

**DOI:** 10.1111/jpi.70174

**Published:** 2026-09-02

**Authors:** Débora B. Constantino, Flavia Baccari, Leonardo Caporali, Valerio Carelli, Giulia Amore, Marijke Gordijn, Toomas Haller, Chiara La Morgia, Anne Landvreugd, Marina Marinelli, David McDaid, Monika Nõmm, Francesco Nonino, Martina Romagnoli, Martino Schettino, Corrado Zenesini, Meike Bartels, Debra J. Skene, Daan R. van der Veen

**Affiliations:** ^1^ Chronobiology Section, Faculty of Health and Medical Sciences University of Surrey Guildford UK; ^2^ IRCCS Istituto delle Scienze Neurologiche di Bologna, UOSI Epidemiologia e Statistica Bologna Italy; ^3^ IRCCS Istituto delle Scienze Neurologiche di Bologna, Programma di Neurogenetica Bologna Italy; ^4^ Department of Biomedical and Neuromotor Sciences (DIBINEM) University of Bologna Bologna Italy; ^5^ IRCCS Istituto delle Scienze Neurologiche di Bologna, UOC Clinica Neurologica Bologna Italy; ^6^ Chrono@Work Groningen the Netherlands; ^7^ Institute of Genomics University of Tartu Tartu Estonia; ^8^ Department of Biological Psychology Faculty of Behavior and Movement Sciences, Vrije Universiteit Amsterdam Amsterdam the Netherlands; ^9^ Care Policy and Evaluation Centre, Department of Health Policy London School of Economics and Political Science London UK

**Keywords:** actigraphy, ageing, light exposure, rest–activity rhythms, sleep

## Abstract

Controlled studies have demonstrated that light supplementation benefits circadian entrainment, sleep and mood. Translating these findings into effective, real‐world usage of light supplementation is, however, challenging because naturalistic and supplemental light exposure are shaped by personal habits and environmental light conditions. Bridging this translation gap is especially critical for older adults, given the reduced ocular light sensitivity associated with natural ageing, which exacerbates low daytime indoor light exposure in urban housing. We therefore set out to investigate the determinants of self‐directed light supplementation in this population, and what the real‐world feasibility and efficacy of daytime light supplementation is. We recruited 202 participants (63–92 years; 139 females) to our interventional, prospective, randomised study in three major EU cities at different latitudes (Amsterdam, Bologna, Tartu). Participants underwent a 2‐week baseline assessment of light exposure, rest–activity patterns and sleep through wearables and daily diaries. Participants were then randomised to either 12 weeks of self‐implemented indoor light supplementation using a lamp placed in their most frequently used room (> 8000 melanopic EDI, *N* = 98) or a no‐intervention group. A subsequent 2‐week reassessment showed that greater naturalistic light exposure was associated with increased daytime activity, consolidated wakefulness, better subjective sleep quality, and reduced metabolic disorder incidence. Individuals with lower naturalistic light exposure self‐initiated earlier and longer indoor light supplementation across seasons, in which supplementation onset before 10:00 was associated with greater daytime activity and lower rest–activity fragmentation. These results confirm that this simple, non‐pharmacological, and low‐cost intervention has broad potential to support healthy ageing in urban settings.

## 1 Introduction

Urbanisation has drastically reduced our exposure to natural daylight and introduced artificial illumination in the evening, both of which impair the appropriate alignment of intrinsic circadian clocks with the naturalistic environment. This circadian misalignment contributes to widespread disturbances in sleep, metabolism and overall health. This is particularly true for older adults, who often experience insufficient light levels during the day [1, 2] and exhibit reduced light sensitivity [3]. Carefully controlled laboratory studies and semi‐controlled home studies have consistently demonstrated that morning light supplementation can mitigate circadian misalignment [4, 5, 6], yet a substantial real‐world evidence base for this remains lacking. A major challenge in translating these promising results of light supplementation protocols from controlled settings into real‐world benefits lies in our limited understanding of the factors that shape the use and timing of self‐administered light exposure and influence its effectiveness, such as age, season, culture, and naturalistic light exposure. To address this gap, the ENLIGHTENme study, a Horizon 2020‐funded consortium (https://www.enlightenme-project.eu/), investigated the feasibility and benefits of self‐implemented light supplementation in the real world. The study monitored timing and quantity of naturalistic light exposure and associated circadian behavioural entrainment and sleep across three European cities (Amsterdam, the Netherlands; Bologna, Italy; and Tartu, Estonia) over five consecutive 3‐month periods in 202 older adults (63–92 years). Participants were divided into an intervention group (*n* = 98) and a control group (*n* = 93), and we assessed how personal and environmental determinants influenced both the adoption and the benefits of the light supplementation.

Light is perceived by rod and cone retinal photoreceptors, as well as by the intrinsically photosensitive retinal ganglion cells (ipRGCs), which are maximally sensitive to short‐wavelength light (460–490 nm) and drive a range of non‐visual responses [7, 8, 9]. Through direct neural pathways, ipRGCs containing melanopsin mediate functions such as the pupillary light reflex and melatonin suppression, while indirectly they regulate sleep–wake cycles, mood, alertness, cognitive functions and other physiological processes [9, 10, 11, 12, 13]. Many of these elicited responses are subject to the timing, intensity, quality and duration of the light stimulus, in which bright daytime exposure is often beneficial, whilst even low‐intensity night‐time exposure can be detrimental [14, 15]. For this reason, bright, blue‐enriched light during the daytime and minimal light at nighttime are recommended to optimise circadian entrainment [15, 16]. These recommendations are consistent with observations that targeted light supplementation, particularly in the morning, can mitigate circadian disruption and improve sleep. However, they also underscore the fact that most people in modern societies receive insufficient light exposure during the day and excessive electric light at night.

The problem of not getting enough daytime light becomes even more pressing with age. Starting around the age of 50, the eye undergoes anatomical and physiological changes that reduce light perception. These include reduced pupil size, loss of rod, cone and ipRGC photoreceptors, and increased thickness and clouding of the lens due to the accumulation of crystalline aggregates and yellow chromophores [17, 18, 19]. As a result, both the quantity and spectral composition of light reaching and detected by the retina are altered, especially in the short‐wavelength range critical for ipRGC activation [20, 21]. Notably, transmission of blue light (460–490 nm) decreases by as much as 72% from childhood (age 10) to old age (age 80) [22]. This reduced transmission of short‐wavelength light impairs photic input to the circadian biological clock in the suprachiasmatic nuclei (SCN) of the hypothalamus, contributing to the circadian disturbances frequently seen in older adults such as increased sleep fragmentation, less consolidated wakefulness, dampening of circadian amplitude, advanced circadian rhythms of core body temperature, melatonin and the sleep–wake cycle [23, 24]. It has been shown that after cataract surgery with transparent lenses, both the sleep–wake cycle as well as the endogenous rhythm of melatonin are delayed, most likely as a result of more light above the threshold passing the lenses in the evening [25].

In an attempt to counteract the effects of low light input, studies have found that improving indoor light conditions can strengthen circadian entrainment, enhance sleep and reduce symptoms of depression and cognitive decline in older adults [4, 6, 26, 27, 28, 29]. These findings, however, primarily stem from controlled experimental studies or interventions in residential care environments, often involving participants with a range of cognitive impairments. Moreover, there is considerable variability in how light therapy is applied across such studies, including differences in timing, duration, intensity and season. It is thus difficult to translate these findings into an unsupervised, real‐world application in which light supplementation is initiated by people themselves in their own homes. Indeed, it is still unclear whether such interventions translate effectively to real‐world conditions, in which individual light exposure is influenced by a complex interplay of personal (e.g., chronotype, age and sex) and environmental (e.g., geographic location, climate, culture and daylight availability) factors [30], which must be considered to understand the ecological validity of light‐based interventions.

The ENLIGHTENme study was an interventional, prospective, randomised, controlled study aimed at investigating the effect of exposure to a specific light supplementation versus no exposure on sleep quality and other indicators of quality of life and wellbeing in a uniquely diverse sample of cognitively healthy older adults living independently across three different latitudes, with data collected throughout all seasons. In this paper, we report: (1) how naturalistic light exposure related to sleep and activity in older adults during the baseline period of the ENLIGHTENme study, (2) which personal and environmental determinants impacted self‐directed indoor light supplementation and (3) the real‐world impact of such determinants on the effects of indoor light supplementation on their rest–activity rhythms and sleep. Understanding these factors under naturalistic, real‐world conditions, across diverse geographic and seasonal contexts, is essential for translating light‐based interventions into an evidence base that supports practical, effective strategies that benefit older adults in their natural environments. This will support initiatives of science‐based advice for the lay public [31].

## 2 Materials and Methods

### 2.1 Study Population

**Figure 1:**
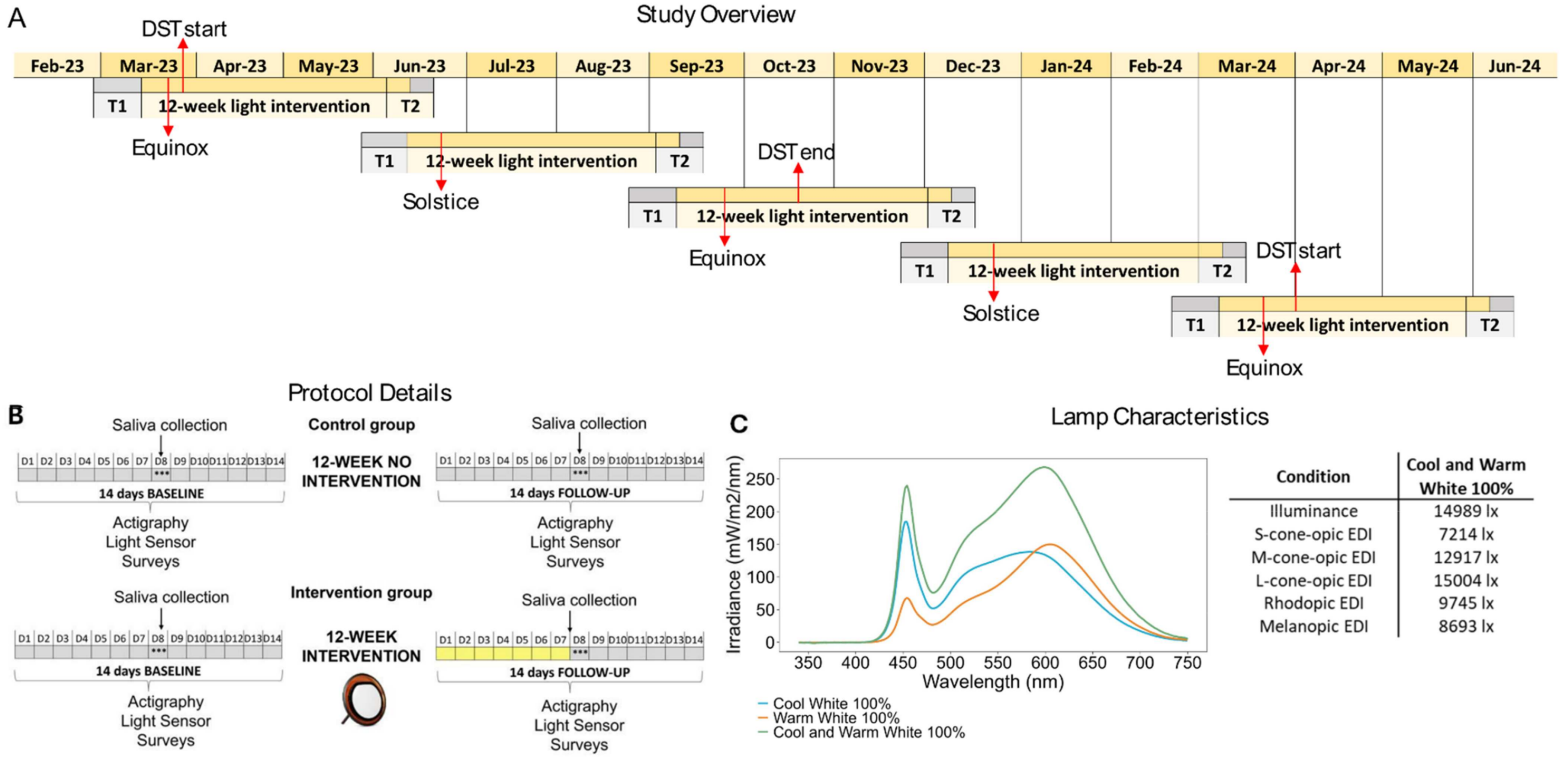
Protocol overview. (A) Study timeline depicting the five waves of data collection strategically timed to avoid equinox, solstice and DST (daylight saving time) changes within or close to recording periods. (B) Experimental protocol for the light intervention and control groups. Grey shaded bars indicate the periods of data collection within each wave. (C) Halo lamp characteristics: Spectral distribution of the cool white (5000 K), warm white (3000 K) and cool and warm white combined (blue); and metrics describing the light intensity with respect to the activation of the five photoreceptors (calculated using the luox platform) [32].

Older adults (*N* = 202, age 63–92 years, *N* = 146 [72%] female) were recruited between February 2023 and February 2024 from specific target districts in three European cities: Amsterdam (52°22′N, 4°53′E), Bologna (44.49°N, 11.34°E), and Tartu (58.378°N, 26.734°E). The target districts were selected based on criteria that included a high level of electric light exposure at night, as well as areas with socio‐economic characteristics that were below the city‐wide average, thus addressing health inequalities in the three cities. The recruitment process involved a wide range of activities, including distributing flyers and posters throughout the cities, placing newspaper and radio advertisements, organising local events by recruiters to promote the study, and advertising in hospitals and healthcare services. Exclusion criteria included the inability to provide informed consent and any health condition that could compromise the participants' data collection throughout the study. Included participants were enroled in five different groups (waves): Wave 1 (February 2023–June 2023), Wave 2 (May 2023–September 2023), Wave 3 (August 2023–December 2023), Wave 4 (November 2023–March 2024) and Wave 5 (February 2024–June 2024) (Figure 1A). Each wave was strategically timed to ensure that major seasonal events, such as equinoxes, solstices and daylight saving time (DST) changes, did not occur within, or in the month prior to, the data recording periods to ensure measurement of stable conditions throughout each study phase. This study was approved by the relevant ethics committee at each of the three recruitment sites. In Bologna, approval was granted by the Ethical Committee AVEC (Area Vasta Emilia Centro; study code n.467_2022‐SPER‐AUSLBO; SIRER ID 4499). In Tartu, approval was granted by the Research Ethics Committee of the University of Tartu (Protocol number 369/T‐18, 17.10.2022). In Amsterdam, approval was obtained from the Medical Ethical Committee at the VU Medical Center and from the Ethical Review Board (VCWE) of the Faculty of Behaviour and Movement Sciences.

### 2.2 Study Design

The study (registered on ClinicalTrials.gov, ID: NCT05676086) employed an interventional, prospective, randomised, before‐after controlled design, conducted in the participants' own homes. Within each wave, participants were randomly assigned to either the light supplementation group (*N* = 98, 63–92 years, 70 female) or the control group (*N* = 93, 66–92 years, 69 female) and took part in a 16‐week study protocol (Figure 1B). The random allocation sequence for randomisation was generated using the ‘Ralloc’ function in Stata software, with a 1:1 ratio, stratified by city.

At the start of the study, each participant completed a 2‐week baseline assessment period. During this phase, participants wore wrist actigraphy watches and pendant light sensors, worn as necklaces (ActLumus, Condor Instruments), to objectively measure individual rest–activity and light exposure patterns. Participants also completed an initial survey and daily activity diaries to provide contextual and behavioural information. On Day 8 of the baseline period, participants collected sequential saliva samples to determine the dim light melatonin onset (DLMO; see Section 2.5), a gold‐standard proxy for SCN timing [33].

Following baseline, participants in the light supplementation group received a commercially available light therapy lamp and initiated a 12‐week light supplementation period (see Section 2.3). The control group continued without light supplementation. At the end of the 12‐week intervention, all participants from both the intervention and control groups completed a 2‐week follow‐up assessment period using the same objective and subjective assessments as in the baseline period. To assess lamp usage and adherence to the recommended timing, those in the light supplementation group were instructed to continue using the lamp throughout the first week of the follow‐up assessment and to report these light exposure times in their daily activity diaries, after which DLMO was again quantified from saliva samples (Day 8).

### 2.3 Light Intervention

Participants assigned to the indoor light supplementation group were given a HALO lamp provided by Lumie (Cambridge, United Kingdom). The HALO lamp utilises both polychromatic warm‐white and cool‐white LEDs to deliver 10 000 lux (> 8000 melanopic EDI) at 20 cm distance at maximum brightness. The cool LED lights have a higher colour temperature of 5000 K, while the warm LED lights have a lower colour temperature of 3000 K. Irradiance and illuminance level, spectral power distribution and melanopic EDI are depicted in Figure 1C. Participants were asked to place the lamp at head height level (e.g., on a table) in the room where they spend most of their time whilst at home (e.g., living room) in order to supplement indoor light exposure; no specific distance from the lamp was prescribed. Participants were not required to follow a strict light exposure schedule; instead, they were instructed to turn on the lamp within 1 h after waking up but not before sunrise, keep it on bright mode for the whole day and turn the lamp off 4 h before their normal sleep time at the latest. They were also asked to use the lamp for at least 2 h, even if not consecutive, throughout the day, for the 12‐week intervention. Usage of the light supplementation was recorded in their daily light diary.

### 2.4 Assessment of Rest–Activity Rhythmicity and Light Exposure

Participants were given an actigraphy watch (ActLumus, Condor Instruments; São Paulo, Brazil) to be worn on their non‐dominant wrist throughout the day and night for 14 days during baseline and during the 14 days of follow‐up, to record their rest–activity patterns. Actigraphy data were recorded in 1‐min epochs and downloaded using the ActStudio software (version 2.1.0). Activity parameters were analysed using the pyActigraphy package (version 1.2) [34]. Actimetry metrics were quantified using nonparametric analyses [35] that provided the following outcomes: Interdaily stability (IS), Intradaily variability (IV), the mean activity count of the 10 consecutive hours with the highest values of a daily profile (M10), the mean activity count of the five least active hours of a daily profile (L5) and relative amplitude (RA). Sleep fragmentation (k_RA_) and activity fragmentation (k_AR_) were also calculated via a probabilistic state transition model [36]. Additionally, we calculated the sleep regularity index (SRI) as a proxy measure of sleep regularity in older adults [37].

To measure individual light exposure, participants were asked to wear a second ActLumus as a pendant necklace over their clothing during the 14 days of baseline and 14 days of follow‐up. When going to bed, they were instructed to place the device on their bedside table to continue measuring light exposure at night. Light recordings were recorded in 1‐min epochs and extracted using the ActStudio software. To characterise light exposure patterns, we conducted the same nonparametric analyses to calculate the measures described for actigraphy data. In addition, we used PyLight (a pyActigraphy module) [38] to calculate total time spent in light levels above 250 melanopic EDI, given that this is the minimum recommended daylight level [15], total time spent above 2000 melanopic EDI as an estimate of time spent outdoors, cumulative melanopic EDI calculated as the sum of 10 consecutive, valid days in each 14‐day recording period, and centre of gravity of light exposure.

### 2.5 Dim Light Melatonin Assessment

Saliva samples were collected by the participants on Day 8 of both the 14‐day baseline and follow‐up assessments (Figure 1B). Participants were instructed to collect 7 saliva samples every hour from 5 h prior to habitual sleep onset until 1 h after habitual sleep onset under dim light conditions (≤ 10 lux) at home. Samples were stored at 4°C until being sent to the institute. Samples were then centrifuged and stored at −80°C until they were shipped on dry ice to the lab of Chrono@Work (the Netherlands). Melatonin was measured by radioimmunoassay (RK‐DSM2, Novolytix, Switzerland), and DLMO was determined as the time when melatonin levels exceeded an absolute concentration of 3 pg/mL for the first time. The limit of detection for the RIA was 0.3 pg/mL with an intra‐assay variation of 7.4% at a low melatonin concentration (mean 2.6 pg/mL, *n* = 14) and 6.3% at a high melatonin concentration (mean = 17.1 pg/mL, *n* = 14). Inter‐assay variation was 30.9% at low melatonin concentration (mean = 0.9 pg/mL, *n* = 16) and 8.7% at high melatonin concentration (mean = 15.3 pg/mL, *n* = 16).

### 2.6 Self‐Reported Variables

Participants completed daily diaries during the 14‐day baseline and follow‐up periods to record the timing of their daily behaviours, including when they slept and used the HALO lamp (follow‐up only).

Assessments were conducted at baseline start (prior to the onset of the light intervention) and at follow‐up (immediately following the conclusion of the intervention). At both time points, the Horne‐Östberg morning/eveningness questionnaire (HO‐MEQ) was used to determine chronotype [39], and the ultra‐short version of the Munich Chronotype Questionnaire (µMCTQ) was used to calculate the midpoint of sleep on weekends (MSFsc) as another marker of chronotype [40]. The Pittsburgh Sleep Quality Index (PSQI) was used to evaluate perceived sleep quality [41]. The Epworth Sleepiness Scale (ESS) was used to estimate sleepiness [42].

### 2.7 Statistical Analyses

In descriptive analyses, data are reported as means and standard deviation (SD), or median and interquartile range (IQR), where appropriate. Normality of data distribution was tested using the Shapiro‐Wilk test and visualised through histograms. To evaluate the univariate association between variables, Chi‐square, Student *t*‐test, Kruskal–Wallis test, and Pearson or Spearman correlations were used. General additive models (GAMs) were used to model the non‐linear relationships between daylength and light supplementation metrics (duration, start and end times).

To investigate the association between light exposure and general metabolic health, participants were assigned to a binary metabolic health status group. Participants who reported any metabolic conditions (high blood pressure, diabetes, obesity and cardiovascular diseases) were grouped as having a metabolic condition, and were compared against those who reported none of these conditions. We used logistic regression models to examine the associations between personal light exposure (as predictor variables) and the binary metabolic health status (as the outcome).

Based on the distribution of baseline cumulative light exposure of all participants, individuals were categorised into three groups: low (the lowest 25th percentile), intermediate (25th–75th percentile), and high (the highest 25th percentile) light exposure. Associations between light exposure categories and actigraphy parameters were evaluated using Kruskal–Wallis, followed by a post hoc Dunn's test with Bonferroni corrections.

To investigate the association between light exposure categories and overall sleep quality, the PSQI score was calculated for participants who answered at least six of the nine items (*N* = 172; 85%), with missing responses imputed using the column‐wise median, stratified by city. Participants with a PSQI score > 5 were classified as having poor sleep quality, while participants with a score ≤ 5 were classified as having normal sleep quality [41]. A logistic regression model was used to examine the association between light exposure groups (as a predictor) and sleep quality (normal vs. poor) as the outcome. The results were presented with odds ratio (OR) and 95% confidence interval (95% CI).

All data extraction and parameter estimation were performed in Python (Version 3.9.12), and statistical analyses were performed in RStudio (Version 2023.03.1+446).

## 3 Results

A total of 202 individuals were recruited (Table 1), with a mean age of 74.7 years (SD: 5.9; range: 63–92); 146 (74%) individuals were female. Participants' mean chronotype was intermediate as indicated by a mean HO‐MEQ score of 16.6 (SD: 3.5; range: 7–23), had a median PSQI of 5.0 (IQR: 3.0–7.2; range: 1–15) and reported no daytime sleepiness at baseline (mean ESS score: 4.8; SD: 3.2). Participants did not report any neurodegenerative disorders, but did report other health conditions, with 56% reporting cardiovascular conditions, 41% musculoskeletal conditions (i.e., arthritis and osteoporosis), and 23% back pain/hernia. Most characteristics did not differ significantly between cities, apart from sex, body mass index (BMI), asthma and inflammatory bowel disease (Table 1).

**Table 1:** Participant characteristics.

	Overall, *N* = 202^a^	Amsterdam, *N* = 14^a^	Bologna, *N* = 66^a^	Tartu, *N* = 122^a^	*p* b
Age (years)	74.7 (63–92)	74.8 (66–92)	74.1 (65–92)	75.1 (63–92)	0.40
Sex					**< 0.01**
Female	146 (74%)	10 (71%)	37 (56%)	99 (84%)	
Male	52 (26%)	4 (29%)	29 (44%)	19 (16%)	
BMI categories					**< 0.01**
Normal	76 (40%)	7 (50%)	36 (56%)	33 (29%)	
Overweight	78 (41%)	5 (36%)	21 (33%)	52 (46%)	
Obese	36 (19%)	2 (14%)	7 (11%)	27 (24%)	
Allergies	32 (16%)	5 (36%)	11 (17%)	16 (14%)	0.11
Asthma	9 (4.6%)	3 (21%)	1 (1.5%)	5 (4.3%)	**0.01**
Migraine	10 (5.1%)	1 (7.1%)	4 (6.1%)	5 (4.3%)	0.66
Epilepsy/seizures	7 (3.6%)	0	0	7 (6%)	0.11
Inflammatory bowel disease	10 (5.1%)	1 (7.1%)	7 (11%)	2 (1.7%)	**0.02**
Liver disease	2 (1%)	0	0	2 (1.7%)	0.60
Kidney disease	6 (3%)	0	1 (1.5%)	5 (4.3%)	0.63
Diabetes	11 (5.6%)	1 (7.1%)	4 (6.1%)	6 (5.1%)	0.79
Back pain/hernia	45 (23%)	1 (7.1%)	15 (23%)	29 (25%)	0.39
Musculoskeletal conditions	81 (41%)	8 (57%)	22 (33%)	51 (44%)	0.20
Cardiovascular conditions	110 (56%)	5 (36%)	35 (53%)	66 (56%)	0.20
Cancer	13 (6.6%)	0	7 (11%)	6 (5.1%)	0.31
Depression	12 (6.1%)	0	5 (7.6%)	7 (6%)	0.80
Fear/Panic attack	7 (3.6%)	1 (7.1%)	0	6 (5.1%)	0.06
Alzheimer	0	0	0	0	N/A
Parkinson	1 (0.5%)	0	1 (1.5%)	0	0.41
Restless legs syndrome	19 (9.7%)	0	3 (4.5%)	16 (14%)	0.09

^a^ Mean (range); *n* (%).

^b^ Fisher's exact test.

Of the 202 participants initially enroled, 11 withdrew and were therefore not assigned to any study group, and the remaining participants were randomly assigned to either the control (*N* = 93) or intervention (*N* = 98) group (Figure 1).

### 3.1 Naturalistic Light Exposure, Timing of Activity and Sleep Quality in Older Adults

We first examined the light exposure of participants in each city and whether this was associated with regional differences in activity timing and sleep. Mean light exposure metrics varied significantly between cities, with participants in Bologna experiencing greater daytime (Light M10, time spent above thresholds [TAT] of 2000 melanopic lux) and nighttime (Light L5) light exposure, whereas participants in Tartu had earlier light exposure times (Onset of light M10 and L5, Timing of light exposure CoG). The time spent above recommended indoor light thresholds (TAT 250) did not differ between cities during baseline (Table 2, upper block). Notwithstanding the different light exposure patterns between cities, we found that most baseline actigraphic variables, including IS, IV, RA, timing and level of activity (M10 and L5), showed no significant differences across the study sites (Table 2, lower block). However, a significant difference was observed for the sleep regularity index (SRI), with participants in Tartu having significantly less regular sleep (lower SRI) compared to Amsterdam and Bologna (Kruskal–Wallis: *p* < 0.01).

**Table 2:** Light exposure and rest–activity metrics across cities.

Variable	Amsterdam (*n* = 14)	Bologna (*n* = 66)	Tartu (*n* = 122)	*p*	Histogram
Light M10 (Melanopic lux)	732 (1171)	863 (2068)	608 (1371)	< 0.01	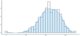
Light L5 (Melanopic lux)	0.50 (1.21)	1.56 (3.37)	1.48 (3.77)	< 0.01	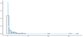
Onset of light M10 (h)	8.47 (1.23)	8.23 (0.9)	8.15 (1.4)	< 0.01	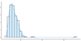
Onset of light L5 (h)	1.18 (1.55)	1.32 (1.37)	1.1 (1.3)	< 0.01	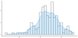
Timing of light exposure CoG (h)	13.3 (0.32)	13.3 (1.09)	12.1 (0.97)	< 0.01	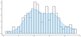
TAT 250 MEDI (min)	2847 (1270)	1917 (1454)	2013 (1908)	0.15	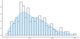
TAT 1000 MEDI (min)	1300 (560)	1132 (1024)	769 (1008)	0.06	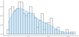
TAT 2000 MEDI (min)	854 (481)	860 (869)	534 (674)	0.03	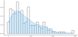
Interdaily stability (IS)	0.53 (0.07)	0.49 (0.13)	0.49 (0.16)	0.15	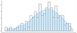
Intradaily variability (IV)	0.51 (0.08)	0.54 (0.19)	0.52 (0.21)	0.82	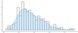
Relative amplitude (RA)	0.67 (0.10)	0.65 (0.19)	0.62 (0.15)	0.50	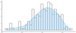
Activity M10	0.96 (0.01)	0.94 (0.05)	0.95 (0.05)	0.09	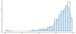
Activity L5	0.19 (0.06)	0.20 (0.13)	0.20 (0.09)	0.62	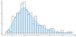
M10 onset (h)	8.33 (0.75)	8.25 (1.54)	8.04 (1.65)	0.50	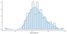
L5 onset (h)	1.33 (1.67)	1.33 (1.31)	1.29 (2.29)	0.53	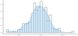
Sleep fragmentation (*k*_RA_)	0.14 (0.02)	0.13 (0.07)	0.13 (0.07)	0.97	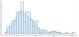
Activity fragmentation (*k*_RA_)	0.01 (0.00)	0.02 (0.01)	0.02 (0.01)	0.26	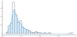
Sleep regularity index (SRI)	81.8 (10.4)	76.5 (11.8)	74.3 (14.6)	< 0.01	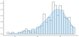

*Note:* Median (IQR); Kruskal–Wallis test.

Abbreviations: CoG = Centre of Gravity, MEDI = Melanopic EDI, TAT = time above threshold.

**Figure 2:**
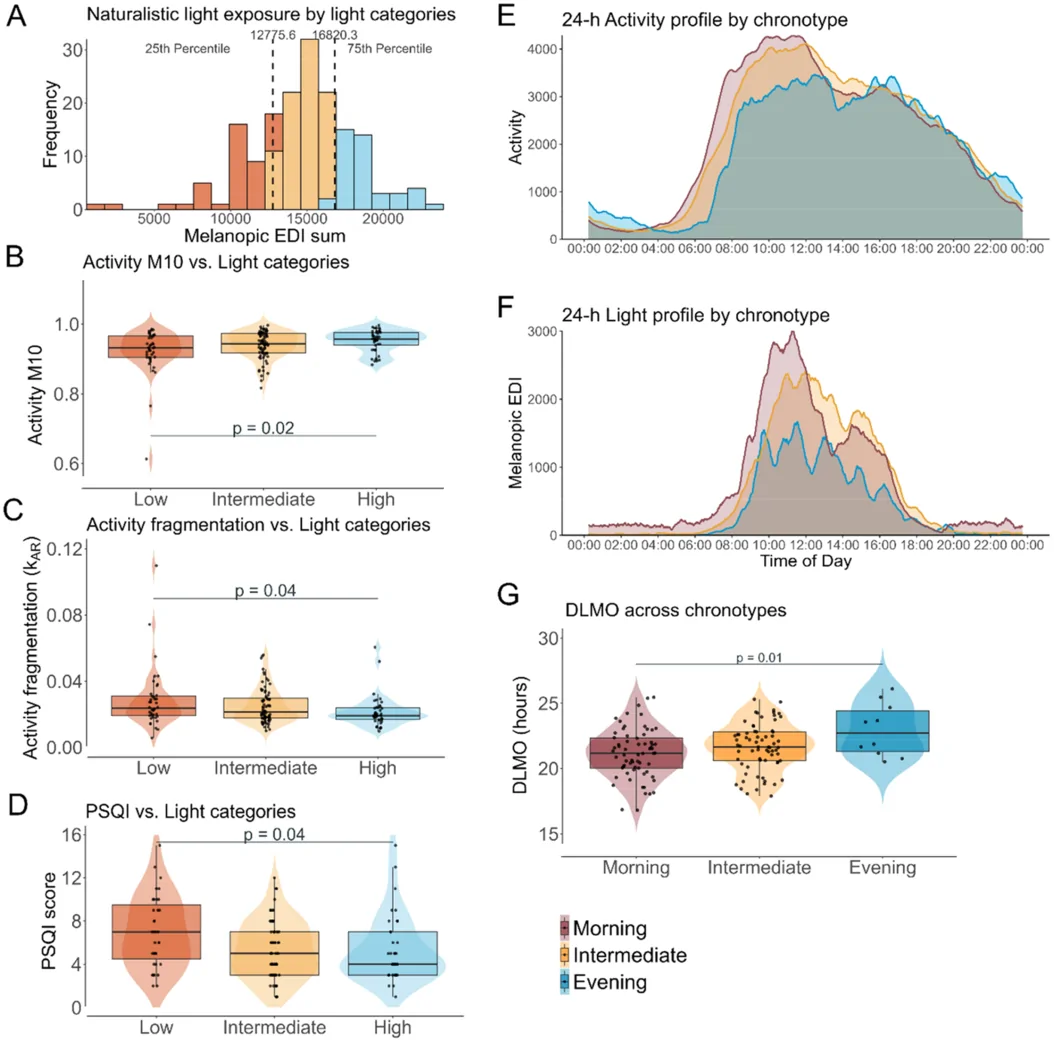
Light exposure levels associated with rest–activity patterns and sleep. (A) Distribution of cumulative melanopic EDI exposure across study participants with median and interquartile range of low (*n* = 42), moderate (*n* = 118) and high (*n* = 42) light exposure. (B) Distribution of M10 activity levels, (C) activity fragmentation (k_AR_), and (D) sleep quality across three light exposure groups. (E) 24‐h distribution of activity levels for each chronotype. (F) 24‐h profile of melanopic Equivalent Daylight Illuminance (EDI) across chronotypes. (G) Distribution of dim light melatonin onset (DLMO) across morning (*N* = 66), intermediate (*N* = 69), and evening (*N* = 10) chronotypes.

We next assessed multi‐collinearity among related variables and examined correlations between light exposure metrics and sleep and activity metrics (Figure S1). We then compared individual naturalistic light exposure patterns and the relationship with an individual's intrinsic circadian timing (DLMO) and rest–activity measures. Participants were categorised into three groups—low, intermediate and high light exposure—based on cumulative light exposure (sum of daily melanopic EDI; Figure 2A). Participants within the low‐light exposure group exhibited significantly lower levels of daytime activity (Kruskal–Wallis: *p* = 0.02) compared to the high‐light exposure group (Figure 2B). Furthermore, those in the low‐light exposure group experienced fewer periods of consolidated wakefulness compared to the high‐light exposure group (Kruskal–Wallis: *p* = 0.04) (Figure 2C). Given the interrelated nature of these factors, we subsequently fitted multivariable models with M10 and K_AR_ (activity fragmentation) as outcomes, controlling for age, sex, season, city and chronotype. Cumulative light exposure and sex were significant predictors of M10 (*β* = 3.9 × 10^−6^, *p* < 0.01; *β* = 0.018, *p* = 0.03, respectively), and light exposure remained a significant predictor of K_AR_ after adjustment for chronotype, age, sex, season and city (*β* = −9.28 × 10^−7^, *p* < 0.01). In terms of sleep quality, participants in the low–light exposure group showed higher PSQI scores (median: 7.0 [4.0–10.0]) than those in the moderate (5.0 [3.0–7.0]) and high (4.0 [3.0–7.0]) light exposure groups (Figure 2D). Compared to participants in the low–light exposure group, those in the high–light exposure group had higher odds of having a good sleep quality (OR = 3.00, 95% CI: 1.16–8.11, *p* = 0.03), while the moderate–light exposure group did not show a significant association. On the other hand, the timing of light exposure (CoG) was not significantly associated with overall sleep quality. These findings highlight the influence of naturalistic light exposure on daily rest–activity patterns, sleep quality and the consolidation of wakefulness.

The timing of light is critical to achieving appropriate circadian entrainment, and the timing of light exposure may in part be driven by chronotype. Consistent with these endogenous preferences, both daily profiles of activity (Figure 2E) and light exposure (Figure 2F) showed clear chronotype‐dependent timing, with the CoG of light exposure and activity occurring earlier in morning types and progressively later in intermediate and evening types (ANOVA, *p* < 0.01). Similarly, DLMO was significantly earlier in morning types compared to evening types (ANOVA, *p* = 0.01), supporting the validity of our measures in real‐world conditions (Figure 2G). Conversely, earlier timing of light exposure (earlier CoG) was also significantly associated with higher odds of belonging to the high‐light exposure group, after controlling for age, sex, season, chronotype, and city (OR = 0.73, 95% CI: [0.65–0.82], *p* < 0.01).

**Figure 3:**
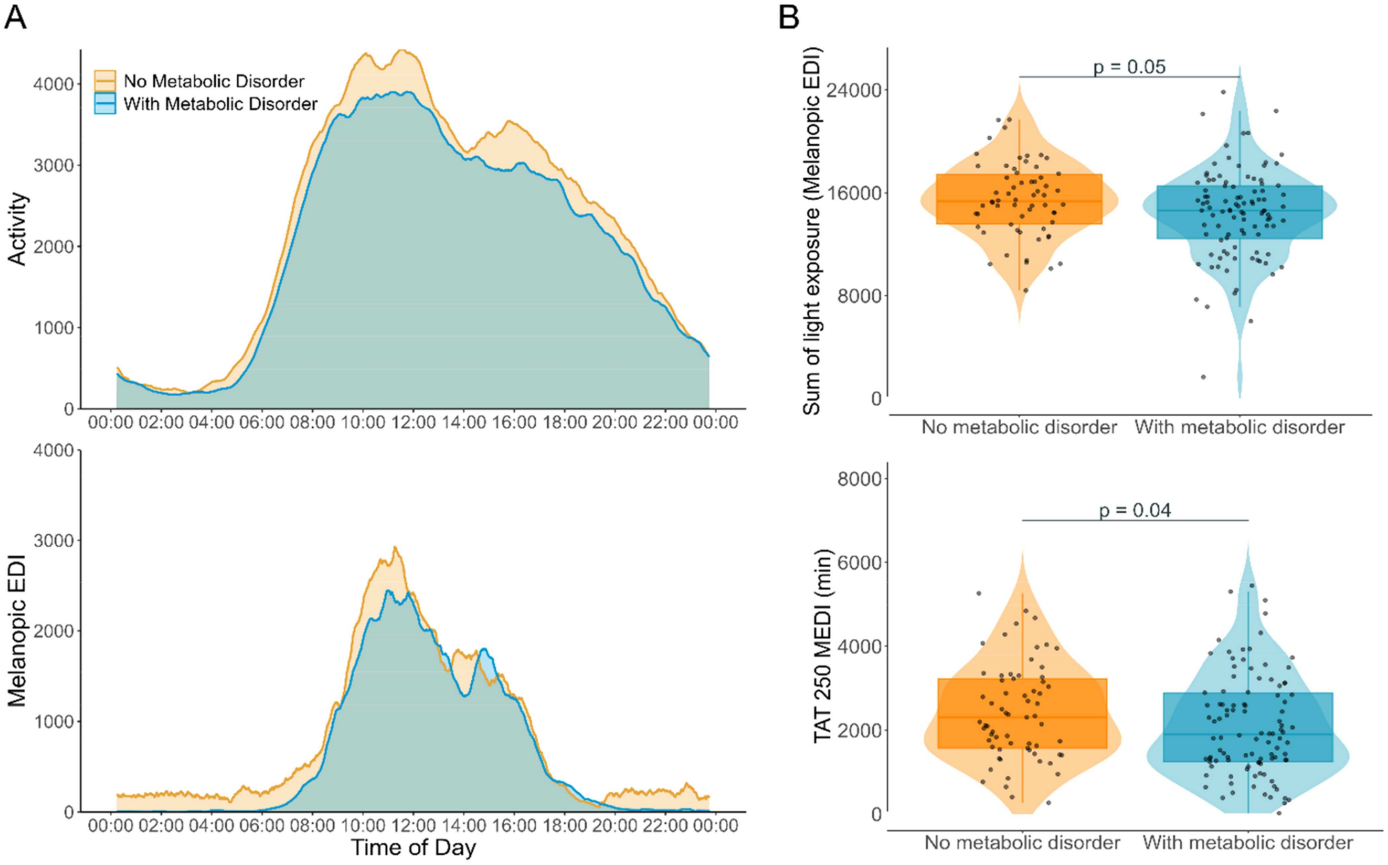
Light exposure patterns across health conditions. (A) 24‐h activity (top panel) and light (bottom panel) profiles for the group with (*N* = 116) and without (*N* = 75) metabolic conditions (high blood pressure, diabetes, obesity and cardiovascular conditions). For each participant, mean activity and light levels were first computed at each minute of the 24‐h day across all valid days of their baseline recording; these individual 24‐h profiles were then averaged within each group. (B) Cumulative melanopic EDI and mean daily time spent above 250 melanopic EDI between participants with no metabolic conditions and participants with metabolic conditions, calculated over a standardised 10‐day baseline window.

Beyond circadian rhythm actigraphy metrics, we also sought to investigate the relationships between personal light exposure and health conditions. We observed that in comparison to participants that did not report metabolic disorders, individuals with metabolic disorders (including high blood pressure, diabetes, obesity, cardiovascular conditions) were exposed to significantly lower levels of total light exposure (Wilcoxon rank‐sum test, *p* = 0.05), and shorter duration of exposure to 250 melanopic lux (TAT 250) (Wilcoxon rank‐sum test, *p* = 0.04) (Figure 3A,B). Indeed, higher cumulative melanopic light exposure was also significantly associated with an increased likelihood of being in the healthy metabolic group (*β* = 1.38 × 10^−4^, *p* = 0.01), after controlling for age, sex, chronotype, daylength and city.

As a final step before examining the effects of light supplementation, we investigated the stability of actigraphy measures between baseline and follow‐up in the control group, to assess the stability of these measures over 16 weeks if no intervention occurred (Figure S2). Due to time‐of‐year variation in environmental light, time spent above (TAT) 2000 melanopic EDI and stability of light exposure (light IS) increased at follow‐up (TAT melanopic EDI: *t* = −4.11, *p* < 0.01; Light IS: *t* = 3.70, *p* < 0.01). However, most actigraphy variables remained stable over the 16‐week protocol, with the only significant difference being a slight decrease in IV (*t* = 3.73; *p* < 0.01) at follow‐up (Table S2). This finding indicates that, despite natural environmental fluctuations, the rest–activity patterns remain largely unchanged over the 16‐week assessment interval.

### 3.2 Environmental and Personal Modifiers of Self‐Administration of Light Supplementation

The control group consisted of 93 participants with a mean age of 74.4 years (SD: 5.4; range: 66–92), and 69 (74%) participants were females. The light supplementation group comprised 98 participants, with a mean age of 75.2 years (SD: 6.5, range: 63–92), and 70 (71%) were females. Importantly, there were no significant differences in any of the assessed sociodemographic characteristics between the control and light supplementation groups (Table S1).

One of the main objectives of the translational approach in this study was to understand the modifiers of self‐determined timing of light supplementation in older adults. Out of the 98 participants in the intervention group, 52 participants (53%) reported HALO daily usage in their activity diaries during the follow‐up assessment. Accordingly, we used this subgroup of 52 adherent participants to examine both environmental and personal determinants of timing of light supplementation.

**Figure 4:**
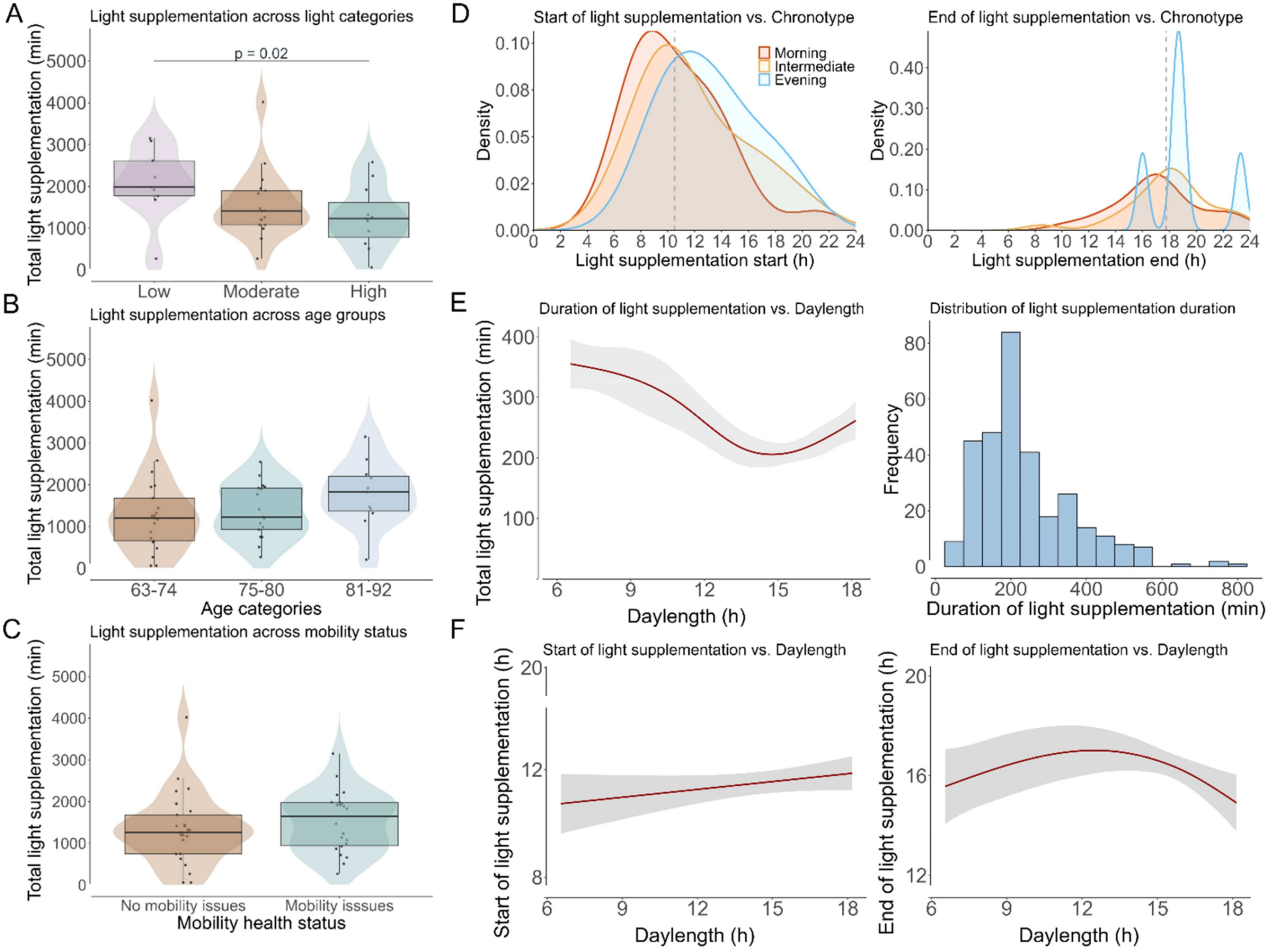
Personal and environmental determinants of indoor light supplementation use. Panels (A–C) show the mean self‐reported daily duration of light supplementation during the first follow‐up week. (A) Total light supplementation across naturalistic light exposure groups: low (*N* = 7), moderate (*N* = 17) and high (*N* = 11) light exposure. (B) Total light supplementation across age groups: 63–74 years (*N* = 21), 75–80 (*N* = 17) and 81–92 (*N* = 11). (C) Total light supplementation between participants with (*N* = 22) and without (*N* = 25) mobility problems. (D) Distribution of timing of light supplementation start and end times across morning (red, *N* = 19), intermediate (yellow, *N* = 23) and evening (blue, *N* = 5) chronotypes. Dashed vertical lines indicate the median start and end times of light supplementation. (E) Relationship between daylength and total duration of light supplementation. (F) Relationship between daylength and the clock time of light supplementation (start and end), with the left panel showing the start time and the right panel showing the end time.

Total duration of light supplementation was found to depend on the overall time spent under natural daylight (TAT > 2000 melanopic EDI). Individuals with low outdoor light exposure exposed themselves to significantly longer (an average of 816 additional min) light supplementation, compared to individuals with greater naturalistic daylight exposure (Kruskal–Wallis: *p* = 0.02) (Figure 4A). We also investigated how age and mobility issues influenced self‐administered light supplementation, as they may impact the opportunity for outdoor light exposure. We did not observe a significant relationship between light supplementation use and age (Kruskal–Wallis: *p* = 0.1) or mobility problems (*t* = 0.76, *p* = 0.1) (Figure 4B,C).

Given that we have previously shown that chronotype was a significant predictor of the timing of light supplementation [6], we also looked at light supplementation timing by chronotype. Although early chronotypes appear to exhibit an earlier onset of light supplementation (Figure 4D), this pattern was not statistically significant (Kruskal–Wallis: *p* = 0.30). Similarly, there was also no significant effect of chronotype on the end time of light supplementation (ANOVA, *p* = 0.46 (Figure 4D).

We did, however, observe that both timing and duration of self‐administered light supplementation varied significantly across seasons. Participants reported a longer duration of self‐implemented light supplementation during the shorter photoperiod of winter, compared to the longer days of summer (*F* = 6.78, *p* < 0.01). Specifically, light supplementation duration increased by > 2‐fold between the shortest and longest days of the year (Figure 4E). As daylength increased and sunrise occurred earlier, participants started their light supplementation later in the day (*F* = 3.31, *p* < 0.01), whereas the end of light supplementation occurred progressively earlier with increasing daylength (*F* = 2.85, *p* = 0.02). Across the observed range (6–18 h of daylength), the start time of light supplementation was delayed by approximately 1.5 h and the end time was advanced by about 1.5 h in long daylength (Figure 4F).

### 3.3 The Benefits of Light Supplementation Depend on the Timing of Light Exposure

**Figure 5:**
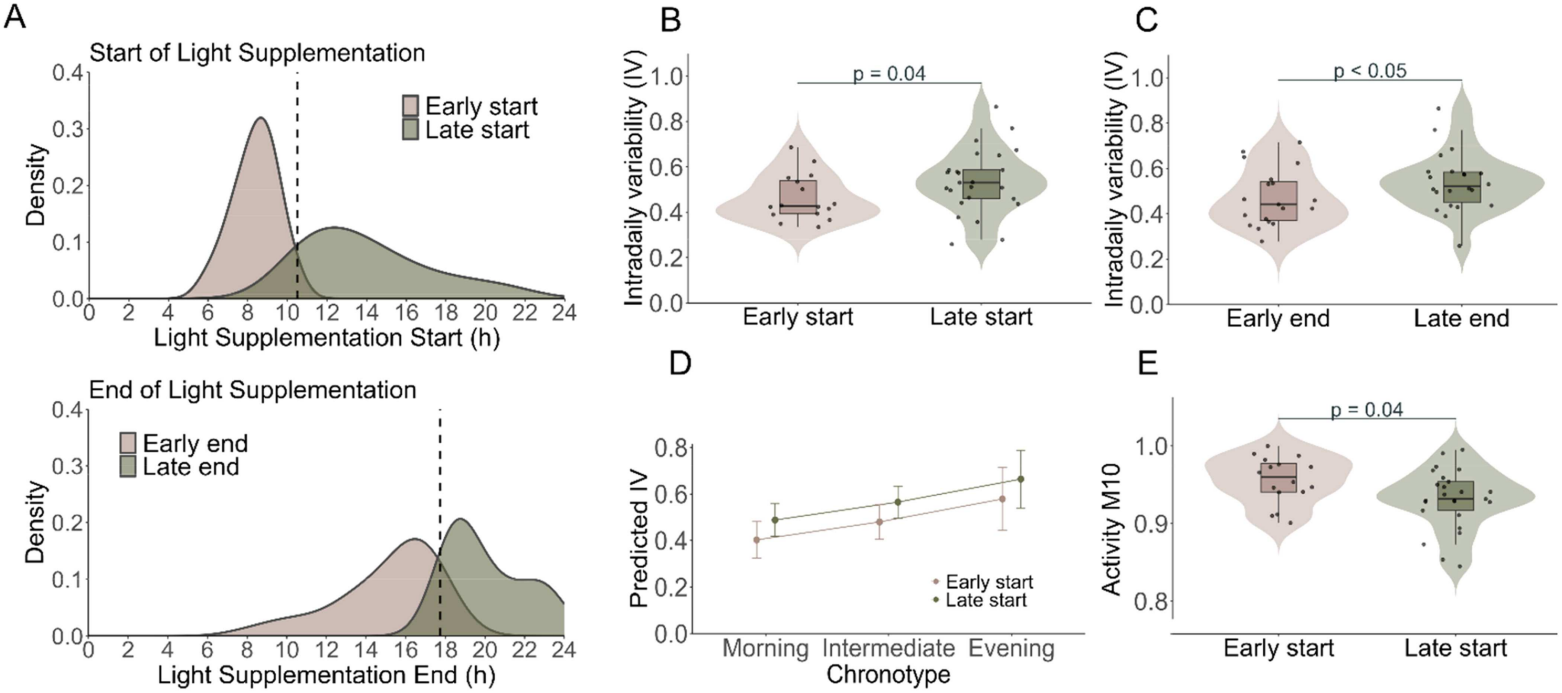
Effects of timing of light supplementation on actimetry outcomes. (A) Histograms illustrating the start (top panel) and end (bottom panel) time of light supplementation. Dashed lines represent the median start and end times of light exposure, which were used as cutoff points to classify participants into early and late exposure. (B) Distribution of intradaily variability across participants that started light supplementation before (early start, *N* = 16) or after (late start, *N* = 24) 10.57 h. (C) Distribution of intradaily variability across participants that ended light supplementation before (early end, *N* = 20) or after (late end, *N* = 19) 17.75 h. (D) Adjusted estimated marginal means (±95% confidence intervals) of IV predicted from a linear model including light exposure group (early vs. late start) and chronotype. (E) Distribution of M10 activity levels across participants that started light supplementation before (early start, *N* = 16) or after (late start, *N* = 24) the median start value of 10.57 h.

Given the substantial impact of the determinants of light supplementation use described above, we further investigated whether the timing of light supplementation itself affected daily rhythms by distinguishing participants who were exposed to light earlier versus later in the day. To do this, we first calculated the median start (10.57 h) and end (17.75 h) times of light supplementation. We then used these median values to distinguish between ‘early’ and ‘late’ supplementation patterns (Figure 5A). Participants who began light supplementation before 10.57 h (*n* = 16) had significantly lower IV (*W* = 128, *p* = 0.05) (Figure 5B) and higher activity M10 (*W* = 276, *p* = 0.04) (Figure 5E) than participants who began after 10.57 h (*n* = 24). Participants who ended light supplementation before 17.75 h (*n* = 20) had significantly lower IV (*W* = 143, *p* = 0.05) compared to those whose light supplementation ended after 17.75 h (*n* = 19) (Figure 5C). Because chronotype is known to influence both light exposure timing and rest–activity metrics, we subsequently adjusted the previous analyses for chronotype (and other possible confounders, such as age, sex and season). Earlier light supplementation onset remained significantly associated with lower IV after adjustment for chronotype (*β* = 0.08, *p* = 0.03), with chronotype also independently associated with IV (*β* = −0.17, *p* = 0.01) (Figure 5D). After adjustment, the association between the start of light supplementation and M10 was no longer significant, indicating that the unadjusted association was largely explained by chronotype. Similarly, the association between the end of light supplementation and IV was no longer significant after adjustment for chronotype. We observed no overall significant differences in outcomes between baseline and follow‐up assessments for either group, or in subsequent between‐group comparisons, without taking into account the timing of light supplementation (Table S3).

## 4 Discussion

Our study is among the few to evaluate the effects of unsupervised, self‐administered light supplementation in real‐world home environments, capturing behavioural patterns in a large cohort of older healthy adults. We provide a comprehensive evidence base that self‐implemented light supplementation is beneficial to circadian entrainment and sleep of community‐dwelling older adults, and that naturalistic light exposure and chronotype are important determinants of the duration and timing of at‐home light usage. The uptake of self‐implemented light supplementation at home is growing, and these results confirm that this simple, non‐pharmacological, and low‐cost intervention has broad potential to support healthy ageing, particularly where daytime light exposure is naturally restricted in urban settings.

The results validate our approach by showing that individuals' chronotypes are strongly associated not only with their intrinsic circadian phase (i.e., DLMO), but also with their behavioural patterns, including the timing of activity and light exposure. As expected, early chronotypes exhibited earlier DLMO and corresponding earlier light exposure and activity profiles, which align with the literature [43, 44, 45]. The observed alignment between circadian phase, behavioural patterns, and light exposure highlights that in real‐world settings, individual differences in circadian biology remained detectable and meaningful in our study population.

We also observed that both the timing and intensity of light exposure were closely linked to sleep and activity patterns. Individuals with higher light exposure showed significantly higher daytime levels of activity, experienced more consolidated (i.e., less fragmented) periods of wakefulness, and reported higher subjective sleep quality compared to those with lower light exposure. This is in line with previous studies that have shown that reduced daytime light exposure is associated with increased sleep–wake fragmentation [6, 46], weaker circadian rhythm amplitude and rest–activity misalignment [47] in cohorts of older men, emphasising the impact of inadequate daylight exposure on rest–activity consolidation. These findings also show that individual differences in naturalistic light exposure were key factors determining how participants used light supplementation.

Although there is substantial evidence showing the negative effects of nighttime light exposure on metabolic outcomes [48, 49, 50], very little is known about the relationship between light exposure during the day and metabolic outcomes. Our observation that low naturalistic light exposure during the day is associated with increased incidence of metabolic conditions supports emerging evidence that insufficient daylight disrupts metabolic regulation [51, 52]. The few published studies suggest a direct link between insufficient daylight exposure and adverse metabolic outcomes. A Brazilian cohort study (Baependi Heart Cohort) objectively measured light exposure and found that greater daytime light exposure and greater contrast between day and night light exposure were associated with reduced risk of metabolic syndrome [53]. This relationship may also be mediated by the timing of daily behaviours, which aligns with the observation that eating at an earlier circadian phase associates with lower BMI, which may be enhanced by earlier daytime schedules [54]. Thus, the health risks are attributed not only to inadequate bright light during the day but also to the balance between daytime and nighttime light exposure. Further research should elucidate the various properties of light (including intensity, duration, and timing) that influence meal timing, metabolic regulation and their mechanisms.

This study identifies the key determinants of self‐directed light‐supplementation use, addressing a topic that has received little prior attention but is crucial to the translation of laboratory studies to real‐world use of light supplementation. Less time spent outdoors was significantly associated with longer duration of light supplementation, which is directly in line with hypotheses that high environmental light exposure acts as a major determinant of light‐seeking behaviours [30]. Seasonal variation in total daylight hours was also a key determinant of self‐directed light supplementation: shorter winter days elicited earlier and prolonged light supplementation, showing that daylength may influence when and how individuals choose to use light interventions. Recently developed conceptual frameworks suggest that light exposure behaviour is influenced by individual needs, psychological traits (including chronotype), personal routines, age, culture, geographical location and environmental factors (such as daylength and temperature) [30, 55, 56]. Our findings converge with these frameworks and indicate that effective strategies to support circadian health in older adults should consider personal traits and environmental characteristics to optimise timing and acceptance of light supplementation.

Importantly, the beneficial effects of light supplementation were strongly dependent on the self‐directed timing of this supplementation. We showed that participants who voluntarily began light supplementation earlier in the morning were significantly more active during the day (higher M10) and had less disrupted rest–activity rhythms (lower IV), consistent with previous findings in controlled settings [6, 57]. Furthermore, those participants who finished light supplementation earlier, thereby reducing evening light exposure, showed lower rest–activity fragmentation (lower IV). These findings are in agreement with Cain et al. [58], who investigated the impact of modern home lighting and found that greater evening light exposure was associated with increased wakefulness after bedtime. Similarly, in Constantino et al. [6], comparing the effects of blue‐enriched versus control white light in the morning and in the evening, we showed that those who had more evening light exposure had increased sleep latency and lowered sleep efficiency. Kim et al. [5] also demonstrated that morning blue‐enriched light improved sleep quality and cognitive function in patients with Alzheimer's disease. Several other studies have shown that inadequate light exposure is associated with higher fragmentation in sleep and rest–activity rhythms in older adults [46, 59]. These findings support the importance of timing in light intervention strategies and, importantly, demonstrate that these effects hold in real‐world settings: early self‐directed light exposure elicits daytime alertness, whereas minimising evening light protects sleep consolidation.

### 4.1 Strengths and Limitations

Our study has several notable strengths. Foremost, light exposure was self‐administered in a real‐world setting over an extended period (12 weeks), enhancing ecological validity and directly addressing the growing uptake of self‐directed use of light therapy lamps in the home. Our recruitment strategy aimed to include older adults across diverse latitudes and all seasons, offering a broad perspective on light's impact under varying geographical and social conditions. We may, however, have enroled more active and educated older adults than the general population, which limits the generalisability of our findings. This generalisability is further constrained by a sex imbalance, as our sample was predominantly female (68%). Crucially, to minimise the burden of an already highly demanding questionnaire battery, we did not collect information regarding postmenopausal status or vasomotor symptoms (VMS), which prevented us from accounting for potential sex‐specific or hormonal factors that might influence sleep and circadian rhythmicity in this cohort.

Moreover, because objective light data were collected only during two discrete 14‐day assessment windows (baseline and follow‐up), rather than continuously throughout the 12‐week intervention, we were not able to disentangle acute (day‐to‐day) from sustained (multi‐week) effects of light exposure; addressing this temporal dimension will require future studies with continuous long‐term monitoring. The real‐world nature of this study means that light exposure was self‐administered and not fully controlled. This is a conceptual strength as it accounts for real‐world conditions, but it does mean that use was highly variable, which constrained the sensitivity of our analyses. Furthermore, this naturalistic approach, where light supplementation was uncontrolled (e.g., variable distance from the light source), may also contribute to the absence of a clear change in actigraphic variables in the overall light intervention group. Moreover, we acknowledge the fact that, despite achieving geographical diversity, there was an imbalance in participant numbers between the three recruitment sites. Finally, the light supplementation protocol was not personalised to individual circadian chronotype profiles; instead, participants were given a deliberately broad opportunity window, which was intended to maximise real‐world uptake, but especially the evening cut‐off may have diluted any chronotype‐related timings of lamp usage. Although we aimed to observe habitual self‐selection and usage patterns, we assessed personal and environmental determinants of light use. Building on this, an important next step will be to develop personalised supplementation strategies that align more closely with individuals' circadian timing and environmental characteristics. Future research may benefit from looking at behavioural interventions, for example, evaluating the benefits of reminder systems and timer‐based systems to help with self‐directed timing for light supplementation.

## 5 Conclusion

In conclusion, our findings suggest that there are critical individual and environmental determinants that influence the benefits of daytime light supplementation. Nonetheless, early administration of light supplementation significantly enhanced activity levels and reduced rest–activity fragmentation in older adults. This work provides real‐world evidence that light is a simple, non‐pharmacological and potentially cost‐effective intervention, which, from a public health policy perspective, has a broad scope to support healthy ageing. Notably, it shows that where daytime exposure to naturalistic light is restricted, individuals often initiate longer self‐directed light supplementation, and that early light supplementation has real‐world benefits for circadian entrainment and sleep. Importantly, future studies should consider how best to integrate determinants of light supplementation and natural light in real‐world settings, as light supplementation is intended to complement, and not replace, exposure to natural daylight. Given the high prevalence of sleep problems and chronic diseases in older adults, these results have immediate implications for clinical and community settings. Light‐based strategies should be integrated into public health policy, clinical care and urban planning. Promoting better light hygiene—including self‐directed light supplementation at home—may offer meaningful benefits to individuals as well as societal benefits by reducing disease burden, particularly as populations continue to age.

## Author Contributions

**Débora B. Constantino:** conceptualisation, methodology, investigation, writing – original draft, writing – review and editing. **Flavia Baccari:** methodology, investigation, writing – review and editing. **Leonardo Caporali:** conceptualisation, methodology, investigation, writing – review and editing. **Valerio Carelli:** conceptualisation, methodology, investigation, writing – review and editing. **Giulia Amore:** investigation, writing – review and editing. **Marijke Gordijn:** conceptualisation, methodology, investigation, writing – review and editing. **Toomas Haller:** conceptualisation, methodology, investigation, writing – review and editing. **Chiara La Morgia:** conceptualisation, methodology, investigation, writing – review and editing. **Anne Landvreugd:** methodology, investigation, writing – review and editing. **Marina Marinelli:** investigation, writing – review and editing. **David McDaid:** conceptualisation, investigation, writing – review and editing. **Monika Nõmm:** methodology, investigation, writing – review and editing. **Francesco Nonino:** conceptualisation, methodology, investigation, writing – review and editing. **Martina Romagnoli:** investigation, writing – review and editing. **Martino Schettino:** investigation, writing – review and editing. **Corrado Zenesini:** investigation, writing – review and editing. **Meike Bartels:** conceptualisation, investigation, writing – review and editing. **Debra J. Skene:** conceptualisation, investigation, writing – review and editing. **Daan R. van der Veen:** conceptualisation, methodology, investigation, writing – original draft, writing – review and editing.

## Conflicts of Interest

D.J.S. is a co‐inventor on issued patents (EP1614441A1 and WO2015/052207A1). M.G. is the director of Chrono@Work, the company performing melatonin analysis on this paper. The other authors declare no conflicts of interest.

## Supporting information


Supporting File

## Data Availability

All data are available in the main text or the Supporting Information. The data sets analysed during the current study are available from the corresponding authors upon reasonable request.
