# Supplementary material for: ENLIGHTENme: Translating the Benefits of Self‐Administered Daytime Light Supplementation to Older Adults in the Real World
Source: J Pineal Res. 2026 Sep 2;78(5):e70174. doi: 10.1111/jpi.70174 (PMC13537858; doi:10.1111/jpi.70174)
Supplement: Supplementary file 1 [file JPI-78-e70174-s001.docx]

**Figure S1. Correlations between light exposure and rest-activity variables.** Pairwise relationships between sleep regularity (Sleep reg), rest-activity metrics (IS: interdaily stability; IV: intradaily variability; RA: relative amplitude; M10: most active 10-hour period; L5: least active 5-hour period), and light exposure variables (time above 250 melanopic lux [TAT 250], time above 2000 melanopic lux [TAT 2000], light during M10 and L5). Scatterplots with fitted distributions are shown in the lower panels, density plots along the diagonal, and Spearman correlation coefficients with significance levels in the upper panels. Asterisks indicate statistical significance (* p < 0.05; ** p < 0.01; *** p < 0.001).

**Figure S2. Stability across measures between baseline (t1) and follow-up (t2).**
